# Nephrotoxic acute kidney injury induced by *Daboia siamensis* venom and its fractions: assessment of *Bcl-2* family expression in *in vivo* and *ex vivo* in rabbit models

**DOI:** 10.1590/1678-9199-JVATITD-2025-0096

**Published:** 2026-07-06

**Authors:** Narongsak Chaiyabutr, Sunutcha Suntrarachun, Suchitra Khunsap, Orawan Khow, Panithi Laoungbua, Taksa Vasaruchapong, Lawan Chanhome, Anudep Rungsipipat, Visith Sitprija

**Affiliations:** 1 Queen Saovabha Memorial Institute, Thai Red Cross Society, Bangkok, Thailand.; 2 Department of Research and Development, Queen Saovabha Memorial Institute, Thai Red Cross Society, Bangkok, Thailand.; 3 Snake Farm, Queen Saovabha Memorial Institute, Thai Red Cross Society, Bangkok, Thailand.; 4 Center of Excellence for Companion Animal Cancer, Department of Pathology, Faculty of Veterinary Science, Chulalongkorn University, Bangkok, Thailand.

**Keywords:** Bcl-2 family, *D. siamensis* venom, Venom fractions, Apoptosis, Acute kidney injury (AKI)

## Abstract

**Background:**

Envenomation by *Daboia siamensis* venom (RVV) can cause acute kidney injury (AKI), but its mechanisms remain unclear. Renal cell death via necrosis and apoptosis is central to AKI progression. Bax and Bcl-2 are key pro- and anti-apoptotic regulators and common markers of apoptosis; however, their roles in RVV-induced renal injury are poorly defined. Therefore, we investigated *Bax* and *Bcl-2* gene expression and their ratio in AKI following the administration of RVV and its venom fractions.

**Methods:**

Kidney tissue samples from intact rabbits (*in vivo*) and the isolated perfused kidney (IPK) model treated with RVV and its venom fractions (PLA₂, MP, LAAO, and PDE) were analyzed for Bcl-2 family expression at both mRNA and protein levels, together with histopathological evaluation. *Bcl-2* and *Bax* mRNA expression was quantified by RT-qPCR using the ΔΔCt method, and protein expression was assessed by immunohistochemical analysis.

**Results:**

Following the administration of RVV and its venom fractions, histopathological analysis revealed extensive tubulonephrosis across all renal tubular segments in both intact kidneys and the IPK model. In contrast, renal tubular necrosis involving all tubular segments occurred in the IPK model after exposure to the RVV, PLA₂ and MP fractions. Immunohistochemical analysis revealed a trend toward increased Bax protein expression and decreased Bcl-2 expression in multiple tubular segments compared to controls in both models. RT-qPCR analysis demonstrated a 1.5-fold upregulation of *Bcl-2* and significant reductions in *Bax* expression and the *Bax/Bcl-2* ratio (*p* < 0.05) in the IPK model relative to controls.

**Conclusions:**

Envenomation with RVV and its venom fractions induced nephrotoxic acute kidney injury by modulating Bax- and Bcl-2-mediated apoptosis in renal tubular epithelial cells, with greater susceptibility observed in intact kidneys and a reduced apoptotic response in the IPK model. These findings suggest that prolonged venom exposure may be partially reversible or may shift cell-death signaling from apoptosis toward necrosis.

## Background

Envenomation caused by venomous snakes is recognized by the World Health Organization (WHO) as a neglected tropical disease and poses a significant public health challenge [1]. Among venomous snakes, *Daboia siamensis (D. siamensis*), a member of the Russell's viper group, is of particular importance. It is found in various Southeast Asian countries and is responsible for numerous clinical envenomation cases, resulting in substantial mortality due to its lethal venom. Acute renal failure (ARF) is one of the most serious complications of envenomation by *D. siamensis*, ans its incidence is increasing significantly [2]. Russell’s viper venom (RVV) consists of a complex mixture of specific enzymatic and non-enzymatic toxins that cause a wide range of pathophysiological events, leading to local and systemic clinical changes. These alterations include intravascular hemolysis [3] and disseminated intravascular coagulation with or without associated microangiopathy [4]. Experimental animal studies have demonstrated that the venom induces hemodynamic alterations resulting in hypotension and circulatory collapse [5], as well as direct nephrotoxicity of the venom [6, 7]. These effects are characterized by worsening kidney function, as evidenced by disturbances in electrolyte and acid-base balance that can occur within hours, days, or even weeks after envenomation [8]. Early administration of antivenom (within 3 to 6 hours after Russell’s viper bite) cannot fully prevent acute kidney injury (AKI) but may lessen the severity of kidney damage [9, 10]. Additional factors contributing to kidney injury include secondary complications, especially oxidative stress and inflammation caused by venom toxins, which may persist despite antivenom treatment and can appear months or even years later [8, 11]. Both clinical and experimental studies show that local factors also contribute to AKI, influenced by inflammatory mediators [12, 13]. 

RVV-induced renal tubular toxicity increases oxidative stress and raises inflammatory cytokine levels without affecting the extracellular matrix. During the acute phase of AKI, urine cytokine profiles shift toward anti-inflammatory dominance within the first two hours after the administration of RVV or its venom fractions, as shown in both *in vivo* and rabbit isolated perfused kidney (IPK) studies [14]. RVV envenomation mainly targets the kidneys, causing lasting renal abnormalities and contributing to chronic kidney disease (CKD) [15]. However, the mechanisms by which RVV causes renal cell injury during the progression of renal failure remain unclear [16]. Renal cell death is central to this process, with necrosis and apoptosis coexisting in AKI despite the challenges associated with distinguishing their pathways. Apoptotic signaling can originate from the cell membrane, the nucleus, or the mitochondria, with the mitochondrial pathway serving as the main determinant of cell survival or death.

There is no consensus on a single mechanism responsible for ARF after viper envenomation, especially regarding RVV-induced mitochondrial toxicity. Our previous work demonstrated impaired mitochondrial respiration and phosphorylation in rat kidneys 24 hours after RVV exposure [17]. In addition to energy production, mitochondria also regulate intracellular signaling and apoptosis. Mitochondrial membrane permeabilization is controlled by Bcl-2 family proteins, which regulate the release of cytochrome c and other apoptotic factors [18, 19]. Pro-apoptotic members (e.g., Bax, Bak) promote cytochrome c release and mitochondrial dysfunction, while anti-apoptotic Bcl-2 keeps the membrane intact by preventing this release [20]. Therefore, the Bax/Bcl-2 expression ratio may be an important indicator of the intrinsic apoptotic process.

The mechanisms underlying this extensive renal cell death observed during envenomation, despite the absence of extracellular matrix damage, remain to be fully elucidated. Furthermore, RVV toxicity is known to promote the generation of reactive oxygen species (ROS), leading to oxidative stress [14]. These processes are considered secondary consequences of snakebite that activate inflammatory pathways and exacerbate renal dysfunction. Because the kidneys receive high blood flow and concentrate circulating toxins, they are particularly susceptible to venom-induced injury. Notably, persistent oxidative damage and inflammatory cell infiltration have been reported even after antivenom administration [8]. Moreover, renal abnormalities may develop months after viper envenomation, posing ongoing challenges for clinical management [21]. While cells are inherently programmed to undergo apoptosis in response to appropriate stimuli, a direct link between venom-induced dysregulation of cell-death pathways and the development of AKI remains to be fully established.

To date, few studies have addressed the role of apoptosis in determining the severity of AKI following snakebite envenomation, as renal cell death in RVV toxicity has largely been attributed to necrosis rather than apoptosis. Consequently, no consensus exists regarding the primary mechanism of ARF after viper bites, particularly concerning the direct cytotoxic effects of RVV on intrarenal tissues.

The objective of this study was to investigate renal cell apoptosis by examining the gene and protein expression of Bcl-2 family members following the administration of RVV and its venom fractions (PLA₂, MP, LAAO, and PDE) in experimental rabbits. Kidney tissues from both *in vivo* and *ex vivo* studies were analyzed using RT-qPCR to quantify *Bcl-2* and *Bax* mRNA expression, and protein expression was assessed by immunohistochemistry. This study enhances our understanding of the underlying pathophysiological mechanisms of AKI during RVV envenomation, which may aid snakebite management and help prevent the onset of early and long-term complications.

## Methods

### Animals

Adult male New Zealand White rabbits weighing 2-3 kg were used for both *in vivo* experiments and *ex vivo* isolated perfused kidney (IPK) studies. All procedures were performed according to a previously described method [14]; however, in this study, kidney tissues from these *in vivo* and IPK experiments were used to evaluate *Bcl-2* family gene expression in nephrotoxic acute kidney injury (AKI) by quantitative real-time PCR (RT-qPCR) and immunohistochemical analysis.

All animal procedures were approved by the Ethics Committee of the Queen Saovabha Memorial Institute Animal Care and Use (QSMI ACUC-03-2016) and conducted in accordance with the guidelines of the National Research Council of Thailand.

### Snakes and venom sample collections

*Daboia siamensis*, native to eastern Thailand, was kept in captivity at the Snake Farm of the Queen Saovabha Memorial Institute (QSMI) in Thailand. Each snake was housed separately in a plastic cage and had unrestricted access to water in the animal care room at the Snake Farm. Once a month, these snakes were fed small rodents based on their weight (10-20% of their body weight). All snakes were maintained under standard conditions with an average ambient temperature of 27 °C and a relative humidity of 75%. Venom from *D. siamensis* snakes was extracted and collected in glass vials. A pool of Russell's viper venom (RVV) was obtained from 14 adult snakes (including both males and females). This venom was lyophilized and stored at -20 °C until use.

### Fractionation of crude *D. siamensis* venom (RVV)

**Figure 1.**
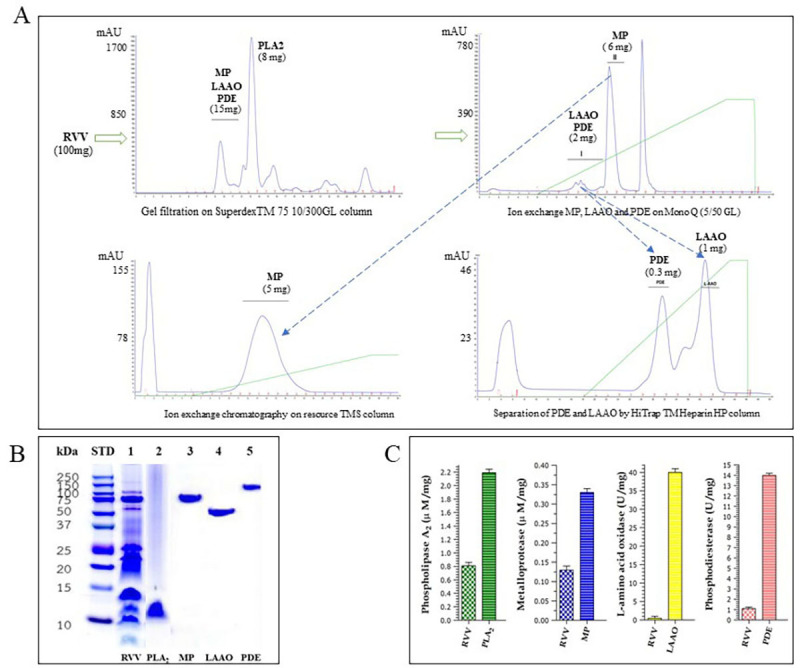
(A) Graphical summary of the purification steps for PLA₂, MP, PDE, and LAAO. Fractionation of crude Russell’s viper venom by gel-filtration chromatography followed by ion-exchange chromatography. (B) Purity of the isolated venom fractions was assessed by 12.5% SDS-PAGE (1 = crude RVV, 2 = phospholipase A_2_, 3 = metalloproteinase, 4 = L-amino acid oxidase, 5 = phosphodiesterase). (C) Enzyme identity and functionality were confirmed by specific enzyme activity assays for PLA₂, MP, LAAO, and PDE.

The fractionation of crude *D. siamensis* venom by gel-filtration and ion-exchange chromatography, along with the determination of the molecular masses of the venom protein fractions and their enzyme activities, are illustrated in Figure 1. A total of 100 mg of lyophilized crude RVV was dissolved in 20 mL of buffer A (50 mM phosphate buffer, pH 6.0) and centrifuged at 10,000 rpm for 5 min at 5 °C. The clear supernatant was applied to a Superdex™ 75 10/300 GL column equilibrated with 0.1 M sodium acetate buffer (pH 6.7). Gel-filtration chromatography was performed using an ÄKTA pure FPLC system at a flow rate of 0.4 mL/min, and 1 mL fractions were collected. Each fraction was assayed for enzymatic activity. Active fractions were pooled, desalted, and concentrated by centrifugal ultrafiltration (Macrosep® 10K; Pall Corp., UK). The fourth peak obtained from gel filtration exhibited phospholipase A₂ (PLA₂) activity.

The first peak from the gel-filtration step was further purified by ion-exchange chromatography on a Mono Q column (5/50 GL; GE Healthcare) pre-equilibrated with buffer A (50 mM Tris-HCl, pH 8.0) and eluted with a linear gradient of buffer B (1 M NaCl) up to 60%. Three distinct protein peaks were obtained. The second peak, which showed metalloproteinase (MP) activity, was further purified using a Resource S column pre-equilibrated with 10 mM sodium phosphate buffer (pH 6.7) and eluted with a linear gradient of 0-0.3 M NaCl.

The first peak from the Mono Q (5/50 GL) column, which exhibited phosphodiesterase (PDE) activity, and the L-amino acid oxidase (LAAO) fraction were further purified using a HiTrap™ Heparin HP column (GE Healthcare) pre-equilibrated with 50 mM Tris-HCl buffer (pH 8.0). Proteins were eluted with a linear gradient of 0-0.5 M NaCl in 10 mM phosphate-buffered saline (pH 7.4) at room temperature. The flow rate was maintained at 0.5 mL/min, and 1 mL fractions were collected.

Protein elution profiles were monitored by absorbance at 280 nm using UNICORN™ 6.3 software. The purification procedures yielded approximately 8.0 mg of PLA₂, 5.0 mg of MP, 1.0 mg of LAAO, and 0.3 mg of PDE (Figure 1A).

### Determination of molecular masses of venom proteins through sodium dodecyl sulfate polyacrylamide gel electrophoresis (SDS-PAGE) 

The molecular masses of the proteins present in crude RVV and its venom gel-filtration (GF) fractions were determined according to the method of Laemmli [22] by 12.5% SDS-PAGE analysis under both reducing and non-reducing conditions. Protein purity was assessed by sodium dodecyl sulfate-polyacrylamide gel electrophoresis (SDS-PAGE). Separating and stacking gels containing 12.5% and 4% acrylamide, respectively, were prepared using a standard gel-casting system and assembled in the electrophoresis apparatus. Snake venom protein samples (10 µL), prepared in sample buffer, along with molecular mass weight standards (5 µL), were loaded onto the gel and electrophoresed in Tris-glycine running buffer (25 mM, pH 8.8) at a constant current of 30 mA until the tracking dye migrated to approximately 0.5 cm from the bottom of the gel. After electrophoresis, the gel was removed from the plates and stained with 0.2% Coomassie Brilliant Blue R-250 for 1 h, followed by destaining in a methanol, acetic acid, and distilled water solution (in a ratio of 25:12.5:62.5, v/v/v). The molecular masses were determined within the mass ranges of 5-50, 50-100, and 100-250 kDa (Figure 1B).

### The enzymatic activities of isolated venom components relating to crude *D. siamensis* venom (RVV)

The isolation of venom components and their enzymatic activities were evaluated in comparison with crude RVV (Figure 1C). Phospholipase A₂ (PLA₂) activity was measured using 3 mM 4-nitro-3-(octanoyloxy) benzoic acid as the substrate. One unit of PLA₂ activity was defined as the amount of enzyme that produced a change in absorbance of 0.1 AU, corresponding to the release of 25.8 nmol of chromophore [23].

Metalloproteinase (MP) proteolytic activity and inhibitor assays were performed using 2% casein in 0.5 M Tris-HCl buffer (pH 8.0) as the substrate, following the method of Anson [24]. The reaction was terminated by the addition of 5% trichloroacetic acid, and hydrolyzed peptides in the supernatant were quantified using the Folin-Ciocalteu method. One unit of proteolytic activity was defined as the amount of enzyme that hydrolyzed casein at an initial rate equivalent to the release of 1.0 µM tyrosine per minute. To assess inhibitor sensitivity, venom samples were pre-incubated with 10 mM EDTA for 10 min prior to activity measurement.

L-amino acid oxidase (LAAO) activity was determined according to the Worthington Enzyme Manual [25], with one unit defined as the amount of enzyme causing an increase in absorbance of 0.001 per min. Phosphodiesterase (PDE) activity was assayed using bis-(p-nitrophenyl) phosphate as the substrate, following the method of Lo et al. [26], and one unit of enzyme activity was defined as the amount of enzyme producing a 0.001 increase in absorbance per minute.

### Animals and experimental design 

A total of 48 adult male New Zealand White rabbits, each weighing 2-3 kg, were used for both *in vivo* and *ex vivo* experiments. All procedures were conducted according to a previously described protocol [14]. The only methodological modification in the present study was the inclusion of histopathological and immunohistochemical analyses, as well as the assessment of Bcl-2 family gene expression in nephrotoxic acute kidney injury, which was quantitatively validated by real-time PCR. Kidney tissues from experimental rabbits were collected from both *in vivo* and isolated perfused kidney (IPK) studies.

### The *in vivo* experimental studies

Twenty-four adult male New Zealand White rabbits were anesthetized with pentobarbital sodium (30 mg/kg body weight, i.v.). The anesthetized rabbits were randomly divided into six groups of four animals each (n = 4 per group). In brief, Group 1 received intravenous injections of 1 mL of 0.15 M NaCl as the control. Group 2 was given intravenous injections of lyophilized crude venom (0.1 mg/kg, i.v.) in 1 mL of 0.15 M NaCl. Group 3 received intravenous injections of the PLA_2_ venom fraction (0.2 mg/kg). Group 4 was administered the MP venom fraction (0.2 mg/kg) intravenously. In Group 5, animals received intravenous injections of the LAAO venom fraction (0.15 mg/kg). Group 6 received intravenous injections of the PDE venom fraction (0.1 mg/kg). The experimental period lasted for 120 min to study the pathophysiological mechanisms of renal function which were described previously [14]. At the end of the study, each animal was euthanized with a high dose of pentobarbital sodium. The left kidney was then carefully removed and immediately excised, with some portions immediately immersed in liquid nitrogen and stored for analysis of *Bcl-2* family gene expression in nephrotoxic acute kidney injury using quantitative real-time PCR (RT-qPCR). The remaining kidney tissue samples were immediately preserved in 10% neutral buffered formalin for histopathological and immunohistochemical analyses. 

Preliminary *in vivo* experiments using a single venom dose of either 0.1 or 0.5 mg/kg body weight (BW) had demonstrated marked dose-dependent differences in outcome. Rabbits injected with 0.1 mg/kg BW developed systemic and renal functional alterations, whereas those receiving 0.5 mg/kg BW generally died within minutes to a few hours after venom administration. This limited survival time prevented adequate evaluation of renal functional changes. Therefore, a dose of 0.1 mg/kg BW was selected for the present study as a compromise between inducing measurable renal injury (confirmed histologically) and maintaining a survival period of at least 2h. Furthermore, intravenous administration of venom at 0.1 mg/kg BW caused only minimal hemodynamic disturbances, whereas higher doses produced progressive hypotension, leading to cardiovascular collapse and, in some cases, rapid death.

### The *ex vivo* experimental studies

Twenty-four adult male New Zealand White rabbits were anesthetized with pentobarbital sodium (30 mg/kg body weight, i.v.). The left kidney was prepared for perfusion after careful dissection following the methods for IPK studies reported in our previous study [14]. Briefly, the experimental trials in the *ex vivo* study of IPK were divided into six groups (n = 4 per group) as follows: 

Group 1: the IPK was treated with 1 mL of 0.15 M NaCl as the control. 

Group 2: the IPK was treated with 1 mL of lyophilized RVV in normal saline (1 mg/mL), added to 100 mL of perfusate in the recirculating system after a 30 min equilibration period. 

Group 3: the IPK was treated with 1 mL of PLA_2_ venom fraction (280 μg/mL), added to 100 mL of perfusate after a 30 min equilibration period. 

Group 4: the IPK was treated with 1 mL of MP venom fraction (280 μg/mL), added similarly after a 30 min equilibration period. 

Group 5: the IPK was treated with 1 mL of LAAO venom fraction (135 μg/mL), added similarly after a 30 min equilibration period. 

Group 6: the IPK was treated with 1 mL of PDE venom fraction (100 μg/mL), added after a 30 min equilibration period. 

Each group underwent 120 min of perfusion for renal function assessment. At the end of the study, parts of the IPK were carefully removed, immediately excised, and stored in liquid nitrogen for evaluating *Bcl-2* family gene expression in nephrotoxic acute kidney injury using RT-qPCR, while other kidney tissue samples were immediately preserved in 10% neutral buffered formalin for histopathological and immunohistochemical analyses.

### Histopathological assessments 

For the morphological studies, renal tissues were randomly collected from four rabbits in each group. Each pre served kidney sample, fixed in 10% neutral buffered formalin (pH 7.2) for 48 h, was dehydrated through a graded ethanol series, processed automatically, and embedded in paraffin wax. Sections (4-6 µm thick) were stained with hematoxylin and eosin (H&E) for general histopathological analyses and periodic acid-Schiff (PAS) staining to analyze glomerular and tubular lesions. All the tissues were examined under a light microscope (Olympus DP-73 camera, Olympus BX53-DIC microscope; Tokyo, Japan). All the changes detected in tissue structures were noted by a board-certified veterinary pathologist, blinded to the treatment groups, who performed light microscopic examinations. The assessment focused on histopathological lesions in the glomeruli, proximal and distal tubules of the renal cortex, and the collecting ducts of the medullary region, analyzing 10 high-power fields per section. Lesions were scored semi-quantitatively as follows: no change (0), mild (1), moderate (2), or severe (3). 

### Immunohistochemical assessments 

Paraffin-embedded rabbit kidney sections (4 µm thick) from each group were heated at 60 °C for 30 min and deparaffinized through a series of xylene and graded ethanol. Immunohistochemical staining was performed to evaluate the expression levels of Bcl-2 family proteins (Bax and Bcl-2) in kidney samples using the labeled streptavidin-biotin (LSAB) method with the EnVision Polymer Detection System (Dako®, Glostrup, Denmark). The sections were subjected to antigen retrieval by autoclaving (121 °C for 10 min) in citrate buffer (pH 6.0). Then, the slides were cooled to room temperature for approximately 20 min, washed in PBS, and the endogenous peroxidase enzyme was blocked with 3% H_2_O_2_ in methanol for 10 min at room temperature. After washing in PBS, non-specific enzymes were blocked with 3% BSA for 30 min and 3% skim milk for 30 min at 37 °C. The kidney tissue sections were incubated overnight at 4 °C in a humidified chamber with monoclonal rabbit anti-Bax antibody (clone E63, code 32503, Abcam, Cambridge, USA) at a 1:200 dilution and monoclonal mouse anti-Bcl-2 protein (clone 3.3.1, Novocastra™, Leica Biosystems) at a 1:100 dilution. Subsequently, the slides were incubated with EnVision™ polymer (Dako, Denmark) for 45 min at room temperature. Peroxidase activity was developed with 3,3'-diaminobenzidine (DAB) for 5 min, and the slides were counterstained with Mayer’s hematoxylin for 2 min. 

The accuracy of each staining protocol was validated using a positive control with canine lymphoma tissue. Rabbit kidney slides without primary antibody served as negative controls. Bax- positive cells showed brown staining in the cytoplasm, while Bcl-2-positive cells were evaluated based on nuclear and intracytoplasmic staining. Ten randomly selected fields (×400) were examined using a Zeiss Primo Star microscope (Zeiss, Oberkochen, Germany) equipped with a Canon EOS 550D camera (Canon, Tokyo, Japan). The percentage of protein expression was calculated as the proportion of positively stained areas over the total area. For protein labeling, quantification was expressed as the percentage of the positive area relative to the total, using ImageJ software (version 1.50i; Bethesda, Maryland, USA). Finally, positive cells were counted in at least 10 low-power fields across three sections per group. Data are presented as the mean ± SD. The staining indices were calculated as follows on the basis of the percentages of the stained nuclei for these two markers: negative: 0 (< 1% positive); weak: 1 (1-25% positive); intermediate: 2 (> 25-75% positive); and strong: 3 (> 75% positive).

### RT-qPCR analysis of apoptosis-regulatory genes 

Extraction of total RNA 

Total RNA was extracted from processed rabbit kidney tissue. Briefly, 10 mg of frozen left kidney tissue was placed into an Eppendorf tube containing 1 mL of TRIzol Reagent (Molecular Research Center, Inc., Cincinnati, Ohio, USA) at 4 °C and vortexed for 20 min. Then, 200 µL of chloroform was added, and the mixture was vortexed for 5 min. The mixture was centrifuged at 12,000 rpm (9,800 × *g*) for 20 min, and the supernatant was collected. An equal volume of isopropanol was added, mixed thoroughly, and incubated at room temperature for 10 min. The solution was centrifuged at 12,000 rpm for 20 min, and the supernatant was discarded. The pellet was then washed with 1 mL of 70% ethanol and centrifuged at 12,000 rpm for 10 min. After removal of the supernatant, the pellet was air-dried at room temperature and stored at −20 °C for later use. 

The RNA precipitate was dissolved in distilled water, and the total RNA concentration was determined using a Qubit RNA assay kit. For quantification, 1 µL of RNA was mixed with 199 µL of Qubit working solution and incubated at room temperature for 5 min. RNA concentration was measured using the Qubit fluorometer with a 200-fold dilution. The purified RNA was stored at −70 °C until further use. 

Synthesis of cDNA

Total RNA was reverse transcribed (RT) into complementary DNA (cDNA) according to the manufacturer’s instructions using the RevertAid™ First Strand cDNA Synthesis Kit (Thermo Scientific, Waltham, MA, USA). A 20 µL reverse transcription reaction mixture containing 1 µg of total RNA and 1 µL of random hexamer primer was adjusted to a final volume of 12 µL with nuclease-free water. The mixture solution was incubated at 65 °C for 5 min and then chilled on ice. Subsequently, 4 µL of 5× reaction buffer, 1 µL of RiboLock RNase Inhibitor (20 U/µL), 2 µL of 10 mM dNTP mix, and 1 µL of RevertAid M-MuLV Reverse Transcriptase (200 U/µL) were added. The reaction was incubated at 25 °C for 5 min, followed by 42 °C for 60 min, and terminated at 70 °C for 5 min. The resulting cDNA samples were stored at −20 °C until further analysis.

Primer design

Oligonucleotide primers for *Bcl-2* and *Bax* were designed for gene expression detection via RT-qPCR and obtained from previously published sequences [27]. Cyclophilin A (*CycA*) was used as a housekeeping gene to normalize target gene expression (Table 1). *CycA* was selected as the reference gene for normalization in RT-qPCR analysis. This selection was further supported by its reported stability in previous studies [28]. Nonetheless, the use of *CycA* was validated under the specific experimental conditions of this study to ensure the accuracy and reliability of gene expression quantification. 

**Table 1.** Primer sequences used for apoptotic genes in sequence-specific detection of the target cDNA for analysis by real-time PCR.

Primer	Nucleotide sequence (5’-3’)	Orientation	Accession number	References
Bax-F Bax-R	TCCACCAAGAAGCTGAGCGAG GTCCAGCCCATGATGGTTCT	Sense Antisense	XM-070063102	Zhao et al. [27]
*Bcl-2*-F Bcl*-*2-R	GGATTGTGGCCTTCTTTGAG CCAAACTGAGCAGAGTCTTC	Sense Antisense	XM-070050072	Zhao et al. [27]
CycA-F CycA-R	CCAACGGCTCCCAGTTCTT ACGTGCTTGCCGTCCAA	Sense Antisense	AF139893	Seol et al. [28]

Quantitative reverse transcription PCR (RT-qPCR)

The mRNA expression levels of *Bcl-2* and *Bax* were measured using quantitative reverse transcription PCR (RT-qPCR) with a LightCycler system (Bio-Rad CFX96 Touch, Berkeley, CA, USA). According to the manufacturer’s instructions, SsoAdvanced Universal SYBR Green Supermix (Bio-Rad, Berkeley, CA, USA) was used to quantify *Bcl-2* and *Bax* gene expression in rabbit samples. Amplification levels were determined using SYBR Green fluorescent dye.

In preliminary experiments, three commonly used housekeeping genes, namely *GAPDH, Hprt1*, and *CycA*, were evaluated. Among these candidates, *GAPDH* and *Hprt1* exhibited greater variability in Ct values across experimental samples, whereas *CycA* showed relatively consistent expression levels, indicating higher stability. Therefore, cyclophilin A (*CycA*) was selected as the reference gene for normalization of RT-qPCR data based on its stable expression under the experimental conditions. 

The final volume of a 10 μL mixture contained 1 μg of cDNA, 5 μL of SsoAdvanced Universal SYBR Green Supermix, and 10 pmol of each complementary primer specific for *Bcl-2* and *Bax* sequences, as well as for the *CycA* sequence as an internal control (housekeeping gene). The reaction was performed under the following conditions: 95 °C for 3 min, followed by 40 cycles of 95 °C for 10 s, 56 °C for 10 s, and 72 °C for 30 s. Melting curve analysis was conducted after amplification to verify the specificity of the PCR product by examining melting temperatures. The melt curve protocol involved heating at 95 °C for 10 s, then increasing the temperature by 0.5 °C every 5 s from 65 °C to 95 °C, with *CycA* used as a housekeeping gene and amplified under the same conditions as above. 

Fluorescent signals were detected at the end of each extension stage. The fluorescence detection threshold was set at the cycle threshold (Ct) corresponding to the inflection point of the fluorescence curve from baseline to exponential phase. Gene expression levels were evaluated using the comparative threshold cycle (Ct) method, also known as the 2^−ΔΔCt^ method. Relative quantification was expressed as fold changes in mRNA expression between groups using the following formula [29].

Fold change = 2^-ΔΔCt^

ΔCt = Ct _target gene_ - average Ct _endogenous control gene_

ΔΔCt = [ΔCt] _target group_ - average [ΔCt]_control group_

### Statistical analysis

All values are expressed as the mean ± SE. Significant differences between the control and each experimental group receiving RVV or its venom fractions were analyzed using one-way ANOVA, followed when appropriate by Bonferroni’s *post hoc* test. A *p*-value < 0.05 was considered statistically significant. When data were not normally distributed and samples were independent, the Mann-Whitney U-test was used to compare the effects of RVV or its fractions on the gene expression of *Bcl-2* family members in renal tissues in both *in vivo* and *ex vivo* studies. All statistical analyses were performed using GraphPad Prism 5 for Windows (GraphPad Software, San Diego, CA, USA).

## Results

### Isolation and enzymatic activity of venom components from crude *Daboia siamensis* venom (RVV)

A total of 100 mg of pooled crude *Daboia siamensis* venom (RVV) was subjected to fractionation to isolate phospholipase A₂ (PLA₂), metalloproteinase (MP), L-amino acid oxidase (LAAO), and phosphodiesterase (PDE). The protein yields obtained were 8 mg for PLA₂, 5 mg for MP, 1 mg for LAAO, and 0.3 mg for PDE (Figure 1A). SDS-PAGE analysis of each venom fraction revealed molecular weights of approximately 13, 90, 60, and 110 kDa for PLA₂, MP, LAAO, and PDE, respectively (Figure 1B). The specific enzymatic activities of all purified venom fractions were substantially higher than those of crude RVV, with increases of 2.6-fold for PLA₂, 2.5-fold for MP, 200-fold for LAAO, and 12.7-fold for PDE (Figure 1C).

### Histopathological findings 

**Figure 2.**
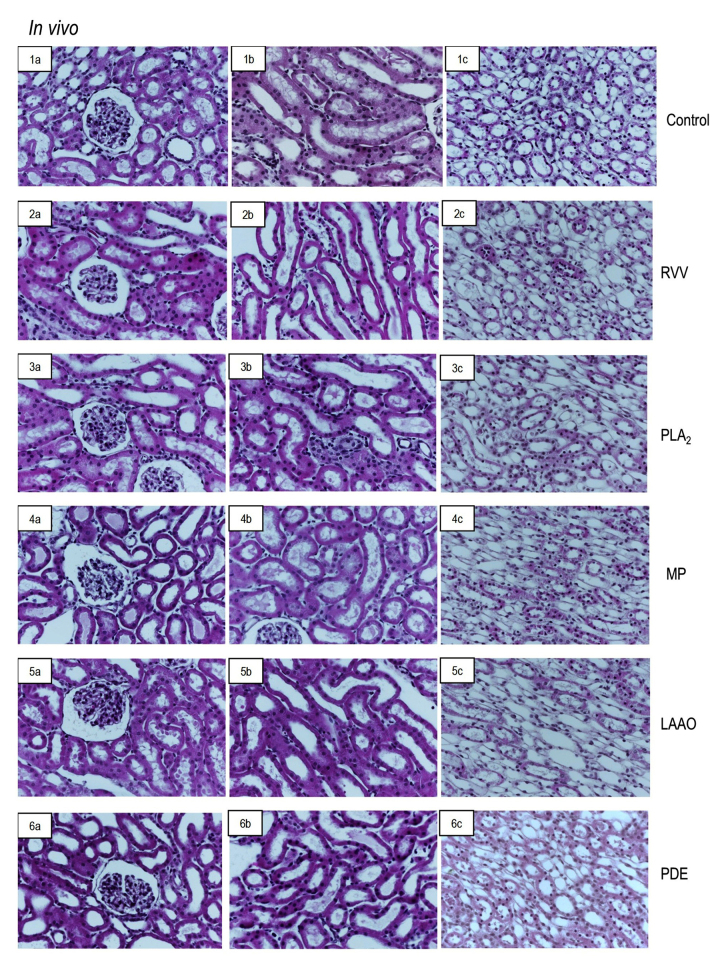
Representative histopathological changes in (a) the glomeruli and tubular regions, including (b) proximal tubules and (c) collecting tubules, of intact rabbit kidneys (*in vivo*) following envenomation. Sections are shown for (1a, 1b, 1c) the control group, (2a, 2b, 2c) crude Russell’s viper venom (RVV), and venom fractions: (3a, 3b, 3c) PLA₂, (4a, 4b, 4c) MP, (5a, 5b, 5c) LAAO, and (6a, 6b, 6c) PDE. H&E staining; ×40 magnification.

**Figure 3.**
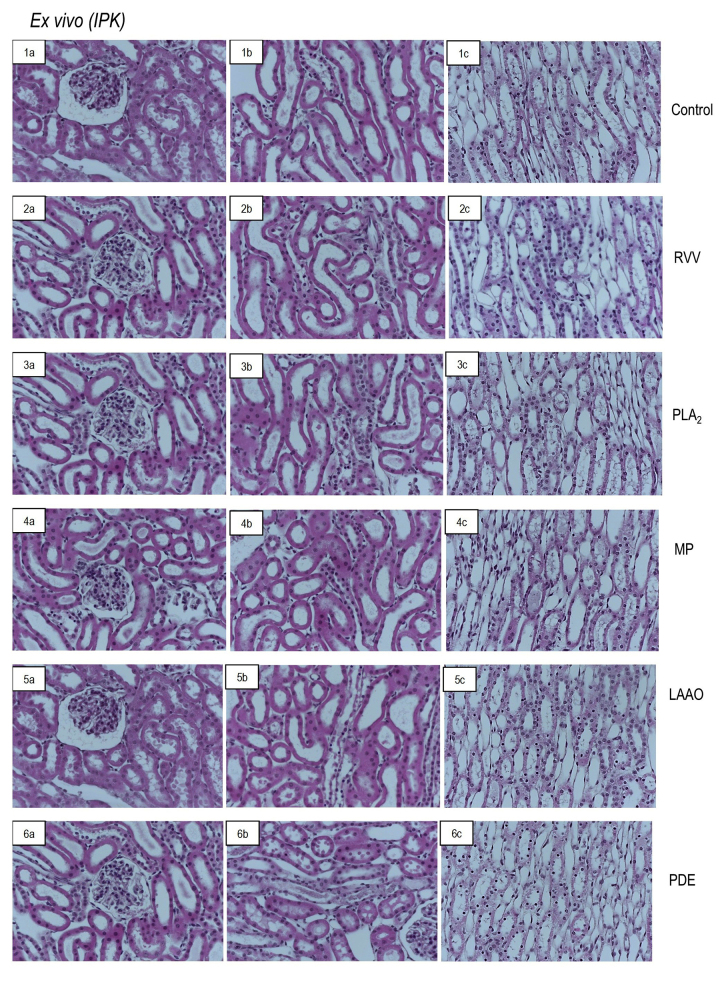
Representative histopathological changes in (a) the glomeruli and tubular regions, including (b) proximal tubules and (c) collecting tubules, of isolated perfused rabbit kidneys (*ex vivo*) following envenomation. Sections are shown for (1a, 1b, 1c) the control group, (2a, 2b, 2c) crude Russell’s viper venom (RVV), and venom fractions: (3a, 3b, 3c) PLA₂, (4a, 4b, 4c) MP, (5a, 5b, 5c) LAAO, and (6a, 6b, 6c) PDE. H&E staining; ×40 magnification.

A comparative scoring system for analyzing histopathological changes in the affected kidneys, both the glomerular and tubular regions, after envenomation with crude RVV venom and its venom fractions (PLA_2_, MP, LAAO, PDE), comparing the intact kidney (*in vivo*) with the IPK, is presented in Table 2. Histopathological images of the intact kidney and the IPK are shown in Figures 2 and 3 , respectively. Histopathological lesions in the renal cortex and medulla were examined and scored under a light microscope by a veterinary pathologist. Each parameter was scored as the average of 10 high-power fields (40×) per sample, focusing on areas with significant tubular damage. Microscopic lesions in the glomerular and tubular regions, including tubular dilatation, tubular cell flattening, tubular cell vacuolization, and tubular cast deposition, were scored on a scale from 0 to 3, where 0= none, 1 = mild (< 25% of the affected area), 2 = moderate (25-50%), and 3 = severe (> 75%).

**Table 2.** Comparative scoring system for analyzing histopathological changes in the glomerular and tubular regions between intact rabbit kidneys (*in vivo*) and isolated perfused kidneys (IPK) following envenomation with crude RVV and its venom fractions (PLA_2_, MP, LAAO, PDE) based on lesion areas observed at 100× magnification (data are shown as mean ± SEM, n = 4 per group).

Lesion category	Study type	Score groups					
		Control	RVV	PLA_2_	MP	LAAO	PDE
Glomerular congestion	Intact kidney IPK	2.90 ± 0.10 0.05 ± 0.09	2.80 ±0.40 0.05 ± 0.05	3.00 ± 0.00 0.24 ± 0.48	3.00 ± 0.00 0	3.00 ± 0.00 0	3.00 ± 0.00 0
Glomerular crystal deposition	Intact kidney IPK	0 0	0 2.23 ± 0.16	0 1.77 ± 1.20	0 1.05 ± 0.94	0 0	0 0
Proximal tubule (tubulonephrosis)	Intact kidney IPK	0 0	1.25 ± 0.17 2.03 ± 0.21	1.40 ± 0.37 2.63 ± 0.62	1.50 ± 0.71 2.70 ± 0.54	1.86 ± 0.58 2.32 ± 0.67	1.73 ± 0.06 1.80 ± 0.13
Proximal tubule (tubulonecrosis)	Intact kidney IPK	0 0	0 0.05 ± 0.10	0 0.15 ± 0.30	0 0.10 ± 0.14	0 0	0 0
Distal tubule (tubulonephrosis)	Intact kidney IPK	0 0	1.10 ± 0.08 1.68 ± 0.56	1.15 ± 0.13 2.45 ± 0.84	1.10 ± 0.42 2.83 ± 0.24	1.93 ± 0.83 2.36 ± 0.74	1.66 ± 0.15 1.75 ± 0.16
Distal tubule (tubulonecrosis)	Intact kidney IPK	0 0	0 0.03 ± 0.05	0 0.35 ± 0.26	0 0.15 ± 0.24	0 0	0 0
Collecting tubule (tubulonephrosis)	Intact kidney IPK	0 0	2.00 ± 0.00 2.43 ± 0.26	2.00 ± 0.00 2.95 ± 0.10	2.00 ± 0.00 2.75 ± 0.50	2.23 ± 0.66 2.54 ± 0.56	1.90 ± 0.31 1.83 ± 0.26
Collecting tubule (tubulonecrosis)	Intact kidney IPK	0 0	0 0.18 ± 0.29	0 0.13 ± 0.25	0 0.18 ± 0.22	0 0	0 0

The values shown in the table represent lesion scores assessed using a semi-quantitative grading system: 0 = no remarkable lesion (NRL); 1 = mild changes; 2 = moderate changes; 3 = severe changes.

In the control group, both the intact kidney and the IPK exhibited normal renal histology with no remarkable lesions in either the glomerular or tubular parts; however, some congestion in the glomerular capillaries was apparent in both kidney models [Table 2; Figure 2 (1a-1c) and Figure 3 (1a-1c)]**.** Intravenous administration of RVV in the intact rabbit kidney groups induced moderate glomerular congestion ***(*** score 2.8), whereas the tubular regions revealed a mild diffuse acute tubulonephrosis in the proximal and distal convoluted tubules and collecting tubules (score 1.10-2.00). The affected tubules exhibited a diffuse cloudy swelling of the cytoplasm with small homogeneous eosinophilic hyaline droplets. Some cells were detached from the tubular basement membrane and contained small, round, dense nuclei [Figure 2 (2a-2c)]**.** Administration of RVV in the IPK group induced mild glomerular congestion (score 0.05) and a moderate crystal deposition (score 2.23), while the renal tubules of the proximal and distal convoluted tubules, as well as the collecting tubules, revealed dilatation and mild-to-moderate diffuse acute tubulonephrosis (score 1.7-2.4), and mild tubulonecrosis (score 0.03-0.18) [Figures 3 (2a-2c)]**.**

Intravenous administration of the venom fraction PLA_2_ *in vivo* induced severe glomerular congestion ***(*** score 3.0) in the intact kidney. Following the administration of the PLA₂ venom fraction, the intact kidney showed mild diffuse acute tubulonephrosis of the proximal and distal convoluted tubules (score 1.15-1.40) and moderate diffuse acute tubulonephrosis of the distal convoluted tubules (score 2.0) [Figure 2 (3a-3c)]**.** Administration of the venom fraction PLA_2_ in the IPK group induced mild glomerular congestion (score 0.24) and a crystal deposition (score 1.77). Regarding the tubular regions of the IPK, the venom fraction PLA₂ induced moderate diffuse acute tubulonephrosis of the proximal and distal convoluted tubules, as well as the collecting tubules (score 2.45-2.95), while mild tubulonecrosis was apparent in the proximal convoluted tubules (score 0.15), distal convoluted tubules (score 0.35), and collecting tubules (score 0.13) in the IPK model [Figures 3 (3a-3c)]**.**

Administration of the venom fraction MP *in vivo* resulted in severe glomerular congestion (score 3.0) in the intact kidney, with no identified crystal deposits in the glomerular capillary lumen, while the proximal and distal convoluted tubules revealed dilatation and mild tubulonephrosis (score 1.1-1.5), and the collecting tubules exhibited moderate tubulonephrosis (score 2.0) [Figure 2. (4a-4c)]. The sections from the rabbit IPK taken 90 min after the administration of the venom fraction MP produced no remarkable lesions with respect to glomerular congestion, while unidentified mild crystal deposits were apparent in the glomerular capillary lumen. The proximal and distal convoluted tubules, as well as the collecting tubules, revealed dilatation and moderate tubulonephrosis (score 2.70-2.83), while these tubular parts showed mild tubulonecrosis (score 0.10-0.18) [Figure 3 (4a-4c)].

Administration of the venom fraction either LAAO or PDE *in vivo* showed severe glomerular congestion (score 3.0) in the intact kidney, while no crystal deposits were apparent in the glomerular capillary lumen of both groups. The proximal and distal convoluted tubules of the intact kidney revealed dilatation and mild tubulonephrosis in the LAAO group (score 1.86-1.93) and the PDE group (score 1.66-1.73), while the collecting tubules had moderate tubulonephrosis (score 1.90-2.22) [Figure 2 (5a-5c) for the LAAO group and Figure 2 (6a-6c) for the PDE group]. In the IPK treated with the venom fraction LAAO, it was observed that dilatation and moderate tubulonephrosis were apparent in the proximal and distal convoluted tubules, as well as the collecting tubules (score 2.32-2.54) [Figure 3 (5a-5c)]. The rabbit IPK treated with the venom fraction PDE revealed dilatation and mild tubulonephrosis in the proximal, distal convoluted tubules, and the collecting tubules (score 1.75-1.83) [Figure 3 (6a-6c)]. 

### Immunohistochemical findings 

The immunohistochemical scores evaluating the effects of the administration of RVV and its venom fractions (PLA_2_, MP, LAAO, and PDE) on the protein expression of the Bcl-2 family in the intact kidney groups and IPK groups are given in Tables 3 and 4, respectively. 

**Figure 4.**
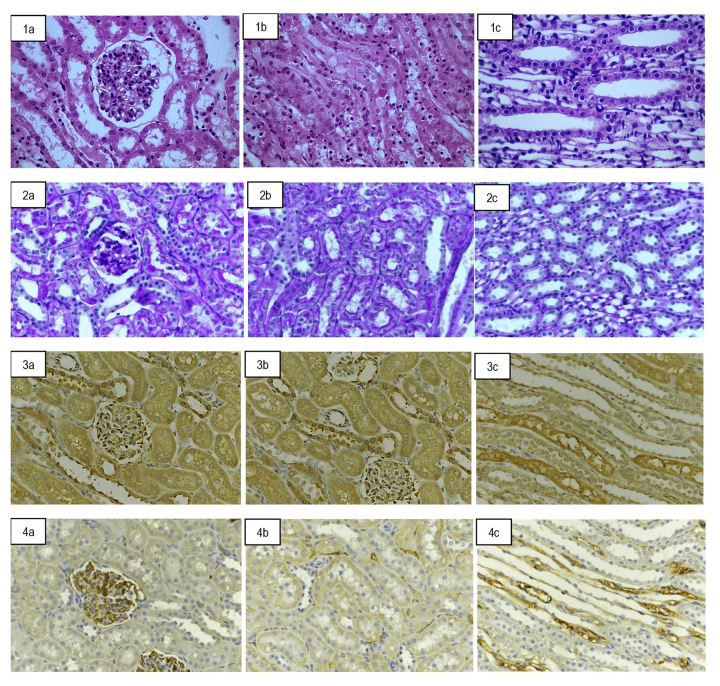
Comparative representative images of the glomerular and tubular regions of intact rabbit kidneys are shown. Histopathological images obtained using H&E staining are presented in panels (1a-1c), while PAS special staining images are shown in panels (2a-2c). Immunohistochemical images demonstrating nuclear and intracytoplasmic Bcl-2 protein expression in tubular epithelial cells are presented in panels (3a-3c), and panels exhibiting brown cytoplasmic staining indicative of Bax-positive tubular epithelial cells are shown in panels (4a-4c).

Immunohistochemical (IHC) images were used to quantify the protein expression of the Bcl-2 family, in comparison with the histopathological changes in the glomerular and tubular regions of intact rabbit kidneys with H&E staining [Figure 4 (1a-1c)], and PAS special staining, which demonstrated bright pink, positive hyaline droplets [Figure 4 (2a-2c)]. Immunohistochemical analysis in Figure 4 (3a-3c) demonstrated Bcl-2 protein expression in tubular epithelial cells, where Bcl-2-positive cells were evaluated based on nuclear and intracytoplasmic staining, while Figure 4 (4a-4c) demonstrated Bax protein expression in tubular epithelial cells, where Bax-positive cells showed brown staining in the cytoplasm. 

**Table 3.** The effects of administration of RVV and venom fractions (PLA_2_, MP, LAAO, and PDE) on the protein expression of the Bcl-2 family (Bcl-2 and Bax) assessed in different segments of the renal tubules in intact rabbit kidneys (*in vivo*) using immunohistochemical analysis (data are shown as mean ± SEM, n = 4 per **group).**

Renal segments	Protein	Groups					
		Control	RVV	PLA_2_	MP	LAAO	PDE
Proximal tubules	Bcl-2 Bax	0.55 ± 0.68^a^ 0.60 ± 0.59^a^	0.03 ± 0.16^b^ 1.00 ± 0.56^a^	0.20 ± 0.41^a^ 1.43 ± 0.81^a^	0.53 ± 0.59^a^ 1.4 ± 0.67^a^	0.50 ± 0.49^a^ 0.45 ± 0.47^a^	0.08 ± 0.13^b^ 0.23 ± 0.26^a^
Distal tubules	Bcl-2 Bax	0.95 ± 0.89^a^ 0.05 ± 0.22^a^	0.20 ± 0.41^a^ 0.12 ± 0.10^a^	0.40 ± 0.47^a^ 0.13 ± 0.34^a^	0.73 ± 0.55^a^ 0.03 ± 0.16^a^	0.50 ± 0.49^a^ 0.45 ± 0.47^a^	0.08 ± 0.13^a^ 0.23 ± 0,26^a^
Collecting tubules	Bcl-2 Bax	0.60 ± 0.50^a^ 0.90 ± 0.72^a^	0.05 ± 0.22^a^ 0.40 ± 0.59^a^	0.05 ± 0.22^a^ 0.73 ± 0.63^a^	0.13 ± 0.33^a^ 0.90 ± 0.54^a^	0.58 ± 0.33^a^ 1.25 ± 0.42^a^	0.03 ± 0.05^a^ 0.28 ± 0.21^a^
Total	Bcl-2 Bax	0.70 ± 0.72^a^ 0.52 ± 0.65^a^	0.09 ± 0.29^a^ 0.51 ± 0.39^a^	0.22 ± 0.41^a^ 0.72 ± 0.59^a^	0.46 ± 0.56^a^ 0.78 ± 0.46^a^	0.53 ± 0.43^a^ 0.72 ± 0.48^a^	0.06 ± 0.10^a^ 0.25 ± 0.24^a^

The values shown in the table are lesion score values that are analyzed in a semi-quantitative manner: 0 = no remarkable lesion (NRL); 1 = mild changes; 2 = moderate changes; 3 = severe changes. Data are shown as mean ± SEM in response to administrations of RVV, PLA₂, MP, LAAO, and PDE in rabbit kidney *in vivo*. Different superscripts (a, b) indicate statistical significance (*p* < 0.05) compared with the control group using analysis of variance (ANOVA) with a *post hoc* Bonferroni test.

**Table 4.** The effects of the administration of RVV and its venom fractions (PLA_2_, MP, LAAO, and PDE) on the protein expression of the Bcl-2 family (Bcl-2 and Bax) were assessed in different segments of the renal tubules in the IPK (*ex vivo*) using immunohistochemical analysis (data are presented as mean ± SEM, n = 4 per group).

Renal segments	Protein	Groups					
		Control	RVV	PLA_2_	MP	LAAO	PDE
Proximal tubules	Bcl-2 Bax	2.17 ± 0.59^a^ 1.16 ± 0.74^a^	1.11 ± 0.63^a^ 2.31 ± 0.64^b^	1.03 ± 0.90^b^ 1.83 ± 0.78^a^	2.32 ± 0.08^a^ 1.93 ± 0.65^a^	2.40 ± 0.22^a^ 2.52 ± 0.45^b^	2.47 ± 0.48^a^ 2.10 ± 0.64^a^
Distal tubules	Bcl-2 Bax	2.53 ± 0.64^a^ 1.00 ± 0.74^a^	0.95 ± 0.71^b^ 0.49 ± 0.69^a^	1.30 ± 0.96^b^ 0.52 ± 0.72^a^	2.50 ± 0.64^a^ 0.30 ± 0.56^a^	2.40+0.22^a^ 2.52 ± 0.45^b^	2.47 ± 0.48^a^ 2.10 ± 0.64^b^
Collecting tubules	Bcl-2 Bax	2.50 ± 0.55^a^ 0.47 ± 0.70^a^	0.92 ± 0.85^b^ 1.46 ± 0.98^b^	0.66 ± 0.51^b^ 0.68 ± 0.83^a^	1.45 ± 0.71^a^ 0.57 ± 0.50^a^	2.62 ± 0.37^a^ 2.60 ± 0.29^b^	2.75 ± 0.25^a^ 2.43 ± 0.48^b^
Total	Bcl-2 Bax	2.44 ± 0.61^a^ 0.83 ± 0.77^a^	0.99 ± 0.74^b^ 1.42 ± 1.08^a^	1.00 ± 0.85^b^ 1.01 ± 0.97^a^	2.09 ± 0.81^a^ 0.93 ± 0.91^a^	2.47 ± 0.27^a^ 2.55 ± 0.39^b^	2.55 ± 0.40^a^ 2.16 ± 0.58^b^

The values shown in the table are lesion score values that were analyzed in a semi-quantitative manner: 0 = no remarkable lesion (NRL); 1 = mild changes; 2 = moderate changes; 3 = severe changes. Data are shown as mean ± SEM in response to the administration of RVV, PLA₂, MP, LAAO, and PDE in the IPK model. Different superscripts (a, b) indicate statistical significance (*p* < 0.05) compared with the control group using analysis of variance with a *post hoc* Bonferroni test.

### **Immunohistochemical score analysis of protein expression of the Bcl-2 family in intact rabbit kidneys (*in vivo*) after administration of RVV and venom fractions (PLA**_2_ **, MP, LAAO, and PDE)**

The immunohistochemical scores of the effects of RVV and its venom fractions on protein expression of the Bcl-2 family (Bcl-2 and Bax) and total expression (Bcl-2 or Bax), assessed in different segments of the renal tubules in the intact kidneys (*in vivo*) are shown in Table 3. The intravenous injection of RVV (0.1 mg/kg) caused decreases in Bcl-2 protein expression across all segments of the renal tubules, with a notable decrease in the proximal tubules as compared to the control group (*p* < 0.05). There was also a significant decrease in the total Bcl-2 expression, calculated from the values of Bcl-2 protein expression across all segments, compared to the control group (Table 3). Bax protein expression in the renal proximal tubules (1.0 ± 0.56) was slightly higher than in the control group (0.6 ± 0.59), although this difference was not statistically significant after RVV administration. 

Intravenous injection of the venom fraction PLA_2_ (0.2 mg/kg) led to a decrease in Bcl-2 protein expression across all segments of the renal tubules, including the total Bcl-2 expression. Administration of PLA₂ caused non-significant, nearly twofold increases in Bax protein expression in the proximal and distal tubules compared to control values. 

Intravenous injection of the MP venom fraction (0.2 mg/kg) slightly decreased Bcl-2 protein expression across all segments of the renal tubules, including the overall Bcl-2 levels. The administration of MP caused non-significant, nearly twofold increases in Bax protein expression in the proximal tubules and total Bcl-2 expression compared to controls. Intravenous injection of the LAAO venom fraction (0.15 mg/kg) showed no change in Bcl-2 or Bax protein expression across any segment of the renal tubules, including total Bcl-2 and Bax level, compared to controls. The intravenous injection of the PDE venom fraction (0.1 mg/kg) reduced Bcl-2 protein expression across all segments of the renal tubules, including the total Bcl-2 level, with a significant decrease in the proximal tubules (*p* < 0.05). Additionally, PDE treatment did not increase Bax protein levels in any segment of the renal tubules, including the total Bax level, compared to the control kidneys. 

### Immunohistochemical score analysis of Bcl-2 family protein expression in the isolated perfused kidney (IPK; *ex vivo*) following administration of Russell’s viper venom (RVV) and venom fractions (PLA₂, MP, LAAO, and PDE)

The immunohistochemical scores of the protein expression of the Bcl-2 family (Bcl-2 and Bax) and total expression (Bcl-2 or Bax), assessed in different segments of the renal tubules in the IPK (*ex vivo*) after administration of Russell’s viper venom (RVV) and its venom fractions (PLA₂, MP, LAAO, and PDE), are shown in Table 4.

Administration of RVV (1 mg/100 mL perfusate), showed significant reductions (*p* < 0.05) in Bcl-2 protein expression across all renal tubule segments, including total Bcl-2. Conversely, there were significant increases (*p* < 0.05) in Bax protein levels in the proximal tubules (+99%), collecting ducts (+213%), and overall Bax (+71%) compared to the control IPK. Administration of PLA₂ (280 μg/100 mL perfusate) also caused significant decreases (*p* < 0.05) in Bcl-2 expression across all renal tubular segments, including the total Bcl-2, compared to the control group. Conversely, Bax expression increased in the proximal tubules (+58%), collecting ducts (+46%), and the total Bax (+21%), while Bax expression in the distal tubules decreased relative to control values. Administration of MP (280 μg/100 mL perfusate) did not cause significant changes in the protein expression of either Bcl-2 or Bax across all tubular segments, including the overall Bcl-2 family expression, compared to the control group. Furthermore, administration of either LAAO (135 μg/100 mL perfusate) or PDE (100 μg/100 mL perfusate) resulted in a non-significant rise in Bcl-2 levels. In contrast, Bax expression significantly increased (*p* < 0.05) across all renal tubular segments, including the overall Bax levels, in both treatment groups compared to the control group.

### Melting curve analysis and RT-qPCR findings

**Figure 5.**
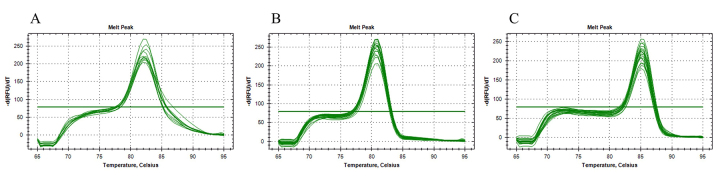
Melting curve analysis of all target genes in the SYBR Green-based qPCR assays. **(A)** *CycA* showing melting temperatures of 82.00-82.50 °C; **(B)** *Bcl-2* showing melting temperatures of 80.50-81.00 °C; and **(C)** *Bax* showing melting temperatures of 85.00-85.50 °C.

According to the RT-qPCR procedure, melting curve analysis in the present study demonstrated specific amplification for all analyzed genes, with each reaction exhibiting a single, sharp peak without primer-dimer formation or non-specific products. The melting temperatures ranged from 82.00-82.50 °C for *CycA* (Figure 5A), 80.50-81.00 °C for *Bcl-2* (Figure 5B), and 85.00-85.50 °C for *Bax* (Figure 5C), were selected for further gene analysis in the SYBR Green-based qPCR assays.

### Relative fold changes in mRNA expression levels of *Bcl-2* and *Bax*, as well as the *Bax/Bcl-2* ratio, were determined by RT-qPCR in intact rabbit kidneys (*in vivo*) following administration of Russell’s viper venom (RVV) and venom fractions (PLA₂, MP, LAAO, and PDE)

The gene expression levels of *Bax* and *Bcl-2* in the treated groups were categorized as high or low expressers based on whether the normalized expression values exceeded or were up to 1-fold relative to the control, respectively, as previously described [29]. Due to the non-normal distribution and high variability of the gene expression data, the Mann-Whitney U test was used to compare each treatment group with the control.

**Figure 6.**
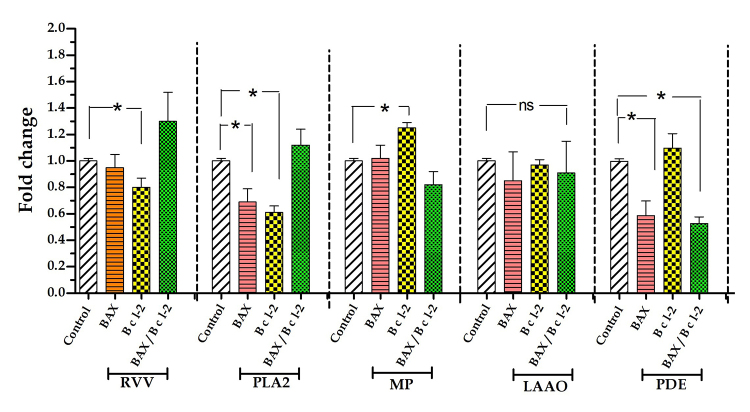
Relative fold changes in mRNA expression levels of *Bcl-2* and *Bax*, as well as the *Bax/Bcl-2* ratio, measured by quantitative real-time RT-PCR in intact rabbit kidneys (*in vivo*) following administration of RVV and its venom fractions (PLA₂, MP, LAAO, and PDE). Data are presented as mean ± SEM (n = 4 per group). Statistical significance was determined using the Mann-Whitney U test; *p < 0.05 compared with the control group; ns: not significant.

In the intact kidney (Figure 6), *Bcl-2* gene expression was notably reduced by 20% compared to the control (*p* < 0.05), whereas *Bax* expression and the *Bax/Bcl-2* ratio showed no significant change following RVV treatment. After PLA₂ administration, both *Bax* and *Bcl-2* gene expression decreased significantly (by 31% and 39%, respectively; *p* <0.05), with a slight increase observed in the *Bax/Bcl-2* ratio. MP administration led to a significant 25% increase in *Bcl-2* expression relative to the control, which decreased the *Bax/Bcl-2* ratio. LAAO administration caused small declines in both *Bax* and *Bcl-2* gene expression, along with a minor reduction in their ratio. PDE administration resulted in a 41% reduction in *Bax* expression, while *Bcl-2* slightly increased, culminating in a significant 47% decrease in the *Bax/Bcl-2* ratio compared to the control.

### **Relative fold change in mRNA expression levels of *Bcl-2* and *Bax*, along with the *Bax/Bcl-2* ratio, was measured using RT-qPCR in IPK (*ex vivo*) following administration of RVV and venom fractions (PLA**_2_ **, MP, LAAO, and PDE)**

**Figure 7.**
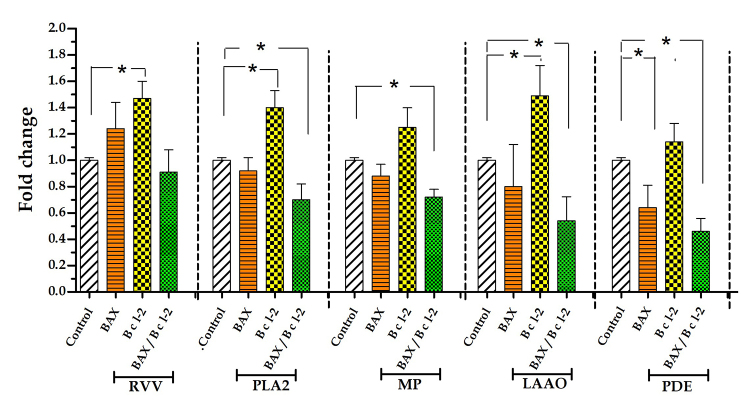
Relative fold changes in mRNA expression levels of *Bcl-2* and *Bax*, as well as the *Bax/Bcl-2* ratio, measured by quantitative real-time RT-PCR in the IPK (*ex vivo*) following administration of RVV and its venom fractions (PLA₂, MP, LAAO, and PDE). Data are presented as mean ± SEM (n = 4 per group). Statistical significance was determined using the Mann-Whitney U test; *p < 0.05 compared with the control group.

Regarding apoptosis-related genes in the IPK model (Figure 7), *Bcl-2* gene expression increased significantly by an average of 47% (*p* < 0.05) after RVV administration, while *Bax* expression showed only a slight rise (+24%). This led to a small decrease in the *Bax/Bcl-2* ratio compared to the control value. Administering PLA₂ led to a notable rise in *Bcl-2* gene expression (+40%, *p* < 0.05), while *Bax* levels did not change. Consequently, the *Bax/Bcl-2* ratio was significantly lower (*p* < 0.05) relative to the control. A similar gene expression pattern was observed after administration of either MP or LAAO. *Bcl-2* levels rose in both, increasing by 24% with MP and 49% with LAAO. Conversely, *Bax* levels fell by 12% in MP and 20% in LAAO. These shifts resulted in significant reductions (*p* < 0.05) in the *Bax/Bcl-2* ratio, 28% for MP and 46% for LAAO, compared to controls. PDE administration led to a notable 36% reduction in *Bax* gene expression (*p* < 0.05) and a modest 14% rise in *Bcl-2* expression. As a result, the *Bax/Bcl-2* ratio was significantly lowered by approximately 54% relative to the control.

## Discussion

Our study describes the action of crude RVV and characterizes the isolation of venom fractions from RVV, identified as PLA₂, MP, LAAO, and PDE. A comparison of the venom fraction activities of the present findings with those previously reported by Chaiyabutr et al. [14] provides strong evidence that these fractions correspond to the same toxins described in that earlier study. Furthermore, our results demonstrate that RVV and its isolated fractions induce renal dysfunction through mechanisms associated with inflammatory responses and oxidative stress [14]. However, the full sequence of toxin actions was not determined; rather, the present study focused on clarifying that AKI resulting from snake envenomation is caused by the disruption or abnormal activation of cell-death pathways. 

AKI is a frequent complication characterized by widespread cell death in multiple renal compartments, including endothelial cells, glomeruli, and proximal and distal tubular epithelial cells [30]. Earlier studies with the rabbit isolated perfused kidney (IPK) indicated that RVV administration directly lowered the glomerular filtration rate (GFR) and reduced sodium reabsorption in both the proximal and distal tubules - results consistent with tubular nephrosis, independent of other extrarenal factors [31]. It has been proposed that the classic view of acute tubular necrosis does not entirely account for how renal nephrotoxins, renal ischemia, or sepsis affect glomerular and tubular function. Evidence from experiments suggests that apoptosis significantly contributes to the development of AKI, reinforcing the concept of renal cell death [32]. 

Although renal cells are the primary targets of RVV and its venom fractions, causing oxidative stress and inducing inflammatory cytokines [14], the underlying mechanisms remain to be fully defined. Reactive oxygen species (ROS) generated during oxidative stress are key mediators of apoptosis and other biological responses [33, 34, 35]; however, the mechanisms underlying apoptosis induced by RVV and its venom fractions remain incompletely elucidated. 

Interestingly, the present study demonstrates that the location of tubular injury varies between types of renal damage after exposure to RVV and its venom fractions, as well as between *in vivo* studies and the IPK model. The distinct histopathological images after envenomation with RVV and the purified venom fractions PLA₂ and MP revealed more extensive and continuous renal tubular necrosis across all parts of the renal tubules in the IPK model. In contrast, such damage was not observed in the intact kidney groups. These differences in histopathological lesion scores between the *in vivo* and IPK groups could be attributed not only to differences in treatment methods or techniques, but also to the prolonged exposure of renal tubular cells to RVV and its venom fractions in the IPK model, allowing for the accumulation of sufficient venom toxins in the renal tissue compartments over time. The transition from apoptosis to necrosis in the cell death pathways might occur in the IPK model during the administration of either RVV or the PLA₂ and MP venom fractions. 

In *in vivo* studies, venom toxins may be redistributed or eliminated from the kidney sites, mitigating harmful effects after envenomation. In addition, in the *in vivo* study, the direct effects of RVV and its fractions may be modulated by systemic feedback mechanisms. Other blood components, such as blood corpuscles, might also help clear apoptotic cells through phagocytosis; these cells are engulfed by phagocytes before membrane rupture, thus shielding nearby tissues from the harmful effects of the release of intracellular contents. Unlike *in vivo* experiments, the IPK model enables the observation of the direct effects of venom and its fractions without the influence of systemic feedback. Since this model lacks blood-borne components such as phagocytic cells, apoptotic cells may not be efficiently cleared by phagocytes or epithelial cells. 

In the IPK model following single administration of the PLA₂ and MP venom fractions, which morphological damage consistent with renal tubular necrosis was observed in multiple tubular segments. This effect can be partly attributed to their hydrolytic enzymatic activity, which increases tubular cell membrane permeability and disrupts membrane integrity, leading to enhanced cytotoxicity and progressive tubular necrosis (Table 2). Damaged cells may undergo secondary necrosis, releasing intracellular contents into the extracellular space, thereby promoting inflammation and exacerbating kidney injury. Necrotic cell death typically occurs in response to severe insults such as snake venom, resulting in plasma membrane rupture, loss of cellular homeostasis, and the release of pro-inflammatory intracellular components [14, 31]. Some researchers suggest that certain apoptotic cells are inherently unstable and may undergo secondary necrosis [36, 37]. However, distinguishing among early apoptosis, late apoptosis, and necrosis remains challenging.

In contrast, in the IPK model, the administration of a single venom fraction LAAO, or PDE administration did not demonstrate the transition from apoptosis to necrosis in the cell death pathways. The LAAO and PDE fractions produced a different pattern of injury, characterized by tubulonephrosis without overt tubular necrosis in the IPK model. LAAO and PDE are considered minor protein components of RVV [38]. The cytotoxicity of LAAO is related to its activity as a homodimeric flavoenzyme that catalyzes amino acid oxidation, generating hydrogen peroxide (H₂O₂), keto acids, and ammonia [39, 40]. The generated H₂O₂ induces oxidative stress, triggering autophagy, apoptosis, and necrosis in target cells [41], and may directly damage cell membranes and vascular endothelium [42]. Similarly, PDE induced tubulonephrosis but not tubular necrosis throughout the 90-min IPK perfusion period, suggesting a mechanism independent of direct membrane disruption. Because PDE hydrolyzes the 3′-phosphoester bond of cAMP and cGMP [43], reduced intracellular cyclic nucleotide levels may contribute to renal cell toxicity. These findings suggest that LAAO and PDE predominantly promote renal tubular injury through apoptosis-related pathways via mechanisms distinct from those of PLA₂ and MP.

The administration of crude RVV in experimental animals has been shown to affect several other intracellular targets; for example, it reduces mitochondrial oxidative phosphorylation and membrane potential in renal cells [17, 44]. However, whether the AKI induced by RVV and its venom fractions is mediated by apoptosis or necrosis, it involves changes in the outer mitochondrial membrane, which is a site for integrating intrinsic and extrinsic pro- and anti-apoptotic signals during cell stress. These changes in coordinated gene expression remain to be clarified.

The present results demonstrate AKI-related gene expression during envenomation with either RVV or its venom fractions, evaluating the expression of *Bcl-2*, *Bax*, and their ratio in kidney samples to assess apoptosis in both intact kidneys and the IPK model. The mechanisms underlying apoptosis are characterized by features such as apoptotic body formation [45] and involve multiple pathways. The extrinsic pathway is initiated by death-receptor ligation, leading to effector caspase activation and irreversible cell death [46]. The intrinsic pathway is regulated by Bcl-2 family proteins, particularly pro-apoptotic Bax/Bak and anti-apoptotic Bcl-2, which control mitochondrial outer membrane permeabilization (MOMP) [47]. Bax/Bak accumulation induces cytochrome *c* release, apoptosome formation with Apaf-1 and caspase-9, and the subsequent activation of caspase-3. Ultimately, cell fate is determined by the balance between pro- and antiapoptotic Bcl-2 family members. However, these pathways do not account for all mechanisms of cell death, as it is well established that apoptosis and necrosis can occur concurrently during the cellular response to injury. Although their morphology, biochemistry, and biological importance differ significantly, distinguishing these pathways can be challenging. 

The effects of RVV and its fractions on cell death may involve the apoptosis pathway, especially the intrinsic pathway controlled by the Bcl-2 family. Regulation within this family might include antioxidant pathways and cell death processes [48]. Members of the *Bcl-2* gene family [49] likely act by disrupting Bcl-2 interactions with mitochondrial antioxidant enzymes like superoxide dismutase [50]. An imbalance between Bcl-2 and Bax can lead to either apoptosis or cell survival [51]. The regulation and effector functions of the intrinsic pathway involve the release of proteins such as cytochrome *c* during mitochondrial outer membrane permeabilization. Nonetheless, the exact mechanism through which pro-apoptotic and anti-apoptotic Bcl-2 proteins initiate apoptosis is not yet fully understood. 

The fold-change results for the gene expression of *Bax* and *Bcl-2*, including their ratio in response to RVV and its fractions during nephrotoxic AKI, differed between the *in vivo* and IPK studies. When analyzing *Bax* and *Bcl-2* individually, *Bcl-2* gene expression was higher than *Bax* in the IPK groups treated with RVV and its venom fractions, although this difference was not statistically significant in the RVV group within the IPK model. The *Bax*/*Bcl-2* ratio in the IPK model served as a dependable marker for the balance between pro-apoptotic and anti-apoptotic proteins. The IPK study showed a consistently lower *Bax*/*Bcl-2* ratio in an anti-apoptotic direction across all groups exposed to RVV and its fractions, with *Bcl-2* being more prevalent; this contrasts with the *in vivo* groups and intact kidney samples, suggesting a shift toward cell survival rather than apoptosis. This finding suggests that a delicate interplay between anti- and pro-apoptotic members of the Bcl-2 family is necessary for the repair of damaged tubular cells after an envenomation insult. Interpreting these responses to envenomation requires further study. 

The protein expression results obtained via immunohistochemical analysis for pro-apoptotic and anti-apoptotic proteins did not match the gene expression analysis of the Bcl-2 family in either the intact or IPK kidneys. Protein expression indicated that apoptotic signaling involved increased levels of Bax (a pro-apoptotic protein) and decreased levels of Bcl-2 (an anti-apoptotic protein) following exposure to RVV and its fractions. This suggests that apoptosis plays a role in AKI development in both the IPK and intact kidney models. Nevertheless, multiple experimental models have demonstrated that Bax overexpression alone can trigger apoptosis in cells both *in vivo* and *in vitro* [52].

In addition to histological alterations, current evidence from immunohistochemical analysis shows high Bax expression and low Bcl-2 expression, indicating that renal epithelial cells, particularly within the proximal tubules and collecting ducts, are highly vulnerable to apoptosis and damage during envenomation in both the IPK and intact kidneys. The differences between these results are likely due to methodological variations, as real-time PCR for gene expression analysis is generally more sensitive and accurate than immunohistochemical analysis for protein detection, as confirmed in many studies.

The present findings suggest that renal cell death induced by RVV or its venom fractions is infrequently due to apoptosis and more often results from regulated necrosis, as shown by histopathological analysis in the IPK model. However, the gene expression levels of *Bax* or *Bcl-2* alone did not reliably indicate apoptosis. Consequently, examining the ratio of *Bax* to *Bcl-2* as a combined marker might offer a more dependable tool for detecting cellular apoptosis.

The differing responses of the *Bax*/*Bcl-2* ratio during the administration of RVV or its venom fractions in the intact kidney and the IPK may result from either a direct interaction of pro-survival Bcl-2, which interferes with Bax/Bak activation, or from Bcl-2 inhibiting Bax/Bak by binding and sequestering BH3-only proteins that would otherwise activate Bax/Bak [53]. Bax has been demonstrated to exist as a monomer in the cytosol [54], indicating that inhibition likely does not occur through direct binding to Bcl-2. One alternative hypothesis suggests that activator BH3-only proteins function as receptors, recruiting pro-apoptotic proteins to the mitochondrial outer membrane, whereas BH3-only proteins with inhibitory roles may bind to pro-apoptotic proteins, preventing their accumulation on the mitochondrial outer membrane (MOM) [53, 54]. The interplay of pro- and anti-apoptotic signals from the Bcl-2 family is crucial in regulating cytochrome *c* release and subsequent apoptotic processes. Nonetheless, the precise mechanism of this inhibition during envenomation remains to be fully understood. 

The administration of RVV and its fractions has been shown to elevate oxidative stress and inflammatory cytokines in kidney tissue in both in vivo studies and the rabbit IPK [14]. This serves as another key indicator of ROS involvement in disrupting mitochondrial membrane potential by opening mitochondrial permeability transition pores, leading to the release of cytochrome c and the activation of caspases 9 and 3, ultimately causing cell death [55]. 

Despite these notable results, this study has several limitations. One limitation is the lack of a comprehensive assessment of the apoptotic pathways involved. Although our findings suggest a potential link between the anti-apoptotic factor Bcl-2 and the pro-apoptotic factor Bax as key modulators of apoptosis that contribute to caspase activation and ultimately renal cell death, a direct evaluation of other apoptotic modulators was not performed. Specifically, components of the intrinsic (mitochondrial) and extrinsic (death receptor-mediated) apoptotic pathways following snake envenomation were not examined. It also remains unclear whether RVV and its venom fractions influence additional regulators of apoptosis, such as transcription factors (e.g., NF-κB and p53) and kinases (e.g., PI3K and JNK), which are known to participate in caspase activation and cell death signaling. Furthermore, alternative apoptotic pathways were not explored. For example, the release of cytochrome c from the mitochondrial intermembrane space leads to its binding with apoptotic protease-activating factor-1 (Apaf-1), forming the apoptosome complex, which subsequently activates caspase-9 and initiates a downstream cascade involving caspase-3, culminating in apoptotic cell death [56, 57]. Future investigations should address these aspects to provide a more comprehensive mechanistic understanding of renal cell death in acute kidney injury caused by snake envenomation.

## Conclusions 

The results of this study, together with evidence from *in vivo* and *ex vivo* experiments, demonstrate that RVV and its venom fractions induce renal cell death while preserving overall tissue structure. Apoptosis appears to play a central role, likely mediated by increased Bax expression and reduced Bcl-2 levels. In the IPK model, prolonged exposure to RVV and its fractions, combined with the absence of phagocytic clearance of apoptotic cells that occurs *in vivo*, may amplify apoptotic responses as a result of sustained toxin exposure to renal tubular cells. When sufficient venom accumulates within renal tissue compartments at critical time points, secondary necrosis may also occur. This study highlights the utility of the IPK model for analyzing the nephrotoxic mechanisms of RVV and its venom fractions in acute kidney injury. Notably, toxicity observed *in vivo* was lower than that predicted by IPK experiments, suggesting that apoptotic responses in the IPK model do not fully recapitulate *in vivo* interactions. Therefore, further experimental studies are required to clarify the mechanisms by which RVV and its venom fractions trigger renal apoptosis and to support the design of more effective translational and clinical studies.

### Abbreviations

AKI: acute kidney injury; Apaf-1: apoptotic protease-activating factor-1; ARF: acute renal failure; Bak: Bcl-2 antagonist/killer 1; Bax: Bcl-2-associated X protein; Bcl-2: B-cell lymphoma 2; CKD: chronic kidney disease; *CycA*: cyclophilin A; GFR: glomerular filtration rate; H&E: hematoxylin and eosin; IPK: isolated perfused kidney; LAAO: L-amino acid oxidase; MOM: mitochondrial outer membrane; MOMP: mitochondrial outer membrane permeabilization; MP: metalloproteinase; PAS: periodic acid-Schiff; PDE: phosphodiesterase; PLA₂: phospholipase A₂; QSMI: Queen Saovabha Memorial Institute; ROS: reactive oxygen species; RT-qPCR: quantitative reverse transcription PCR; RVV: *Daboia siamensis* venom; SEM: standard error of the mean. 

## Data Availability

All data generated or analyzed during this study are included in this article.

## References

[B1] World Health Organization (2023). Snakebite envenoming.

[B2] Alvitigala BY, Gooneratne LV, Gnanathasan CA, Wijewickrama ES (2025). Snakebite-associated acute kidney injury in South Asia: narrative
review on epidemiology, pathogenesis and management. Trans R Soc Trop Med Hyg.

[B3] Sofyantoro F, Sudaryadi I, Yudha DS, Raharjo S, Purwestri YA, Nuringtyas TR (2025). Renal toxicity in snakebite envenomation: insights into
pathophysiology, risk factors, and management strategies. J Current Sci Tech.

[B4] Lu Q, Clemetson JM, Clemetson KJ (2005). Snake venoms and hemostasis. J Thromb Haemost.

[B5] Tungthanathanich P, Chaiyabutr N, Sitprija V (1986). Effect of Russell's viper (Vipera russelli siamensis) venom on
renal hemodynamics in dogs. Toxicon.

[B6] Chaiyabutr N, Vasaruchapong T, Chanhome L, Rungsipipat A, Sitprija V (2014). Acute effect of Russell's viper (Daboia siamensis) venom on renal
tubular handling of sodium in isolated rabbit kidney. Asian Biomed.

[B7] Chaiyabutr N, Chanhome L, Vasaruchapong T, Laoungbua P, Khow O, Rungsipipat A, Sitprija V (2020). The pathophysiological effects of Russell's viper (Daboia
siamensis) venom and its fractions in the isolated perfused rabbit kidney
model: A potential role for platelet activating factor. ToxiconX.

[B8] Sunitha K, Hemshekhar M, Thushara RM, Sebastin Santhosh M, Shanmuga Sundaram M, Kemparaju K, Girish KS (2015). Inflammation and oxidative stress in viper bite: An insight
within and beyond. Toxicon.

[B9] Aye KP, Thanachartwet V, Soe C, Desakorn V, Thwin KT, Chamnanchanunt S, Sahassananda D, Supaporn T, Sitprija V (2017). Clinical and laboratory parameters associated with acute kidney
injury in patients .with snakebite envenomation: a prospective observational
study from Myanmar. BMC Nephrol.

[B10] Hung DZ, Yu YJ, Hsu CL, Lin TJ (2006). Antivenom treatment and renal dysfunction in Russell’s viper
snakebite in Taiwan: a case series. Trans R Soc Trop Med Hyg.

[B11] Sebastin Santhosh M, Hemshekhar M, Thushara RM, Devaraja S, Kemparaju K, Girish KS (2013). Vipera russelli venom-induced oxidative stress and hematological
alterations: amelioration by crocin a dietary colorant. Cell Biochem Funct.

[B12] Vahed S Zununi, Khatibi SM Hosseiniyan, Ardalan M (2025). Canonical effects of cytokines on glomerulonephritis: A new
outlook in nephrology. Med Res Rev.

[B13] Anders HJ, Kitching AR, Leung N, Romagnani P (2023). Glomerulonephritis: immunopathogenesis and
immunotherapy. Nat Rev Immunol.

[B14] Chaiyabutr N, Noiprom J, Promruangreang K, Vasaruchapong T, Laoungbua P, Khow O, Chanhome L, Sitprija V (2024). Acute phase reactions in Daboia siamensis venom and
fraction-induced acute kidney injury: the role of oxidative stress and
inflammatory pathways in in vivo rabbit and ex vivo rabbit kidney
models. J Venom Anim Toxins incl Trop Dis.

[B15] Herath HM, Wazil AW, Abeysekara DT, Jeewani NDC, Weerakoon KGAD, Ratnatunga NVI, Bandara EHCK, Kularatne SAM (2012). Chronic kidney disease in snake envenomed patients with acute
kidney injury in Sri Lanka: a descriptive study. Postgrad Med J.

[B16] Resiere D, Mehdaoui H, Neviere R (2022). Inflammation and oxidative stress in snakebite envenomation: A
brief descriptive review and clinical implications. Toxins.

[B17] Julsukon A (1995). Effects of Russell's viper venom on renal functions in
selenium-supplemented rats.

[B18] Reed J, Jurgensmeier J, Matsuyama S (1998). Bcl-2 family proteins and mitochondria. Biochim Biophys Acta.

[B19] Scarlett JL, Murphy MP (1997). Release of apoptogenic proteins from the mitochondrial
intermembrane space during the mitochondrial permeability
transition. FEBS Lett.

[B20] Yang J, Liu X, Bhalla K, Kim CN, Ibrado AM, Cai J, Peng TI, Jones DP, Wang X (1997). Prevention of apoptosis by Bcl-2:release of cytochrome c from
mitochondria blocked. Science.

[B21] Girish KS, Kemparaju K (2011). Overlooked issues of snakebite management: time for strategic
approach. Curr Top Med Chem.

[B22] Laemmli UK (1970). Cleavage of structural proteins during the assembly of the head
of bacteriophage T4. Nature.

[B23] Holzer M, Mackessy SP (1996). An aqueous endpoint assay of snake venom phospholipase
A2. Toxicon.

[B24] Anson ML (1938). The Estimation of pepsin, trypsin, papain, and cathepsin with
haemoglobin. J Gen Physiol.

[B25] Worthington Enzyme Manual (1977). L-amino acid oxidase.

[B26] Lo TB, Chen YH, Lee CY (1966). Chemical studies of Formosan cobra (Naja naja atra) venom. Part
I. Chromatographic separation of crude venom on CM-Sephadex and preliminary
characterization of its components. J Chin Chem Soc.

[B27] Zhao S, Yu H, Du N (2015). Experimental study of doxorubicin interventional chemotherapy in
the treatment of rabbit VX2 renal transplantation carcinoma. Int J Clin Exp Med.

[B28] Seol D, Choe H, Zheng H, Jang K, Ramakrishnan PS, Lim TH, Martin JA (2011). Selection of reference genes for normalization of quantitative
real-time PCR in organ culture of the rat and rabbit intervertebral
disc. BMC Res Notes.

[B29] Schmittgen TD, Livak KJ (2008). Analyzing real-time PCR data by the comparative C(T)
method. Nat Protoc.

[B30] Chaiyabutr N, Chanhome L, Vasaruchapong T, Laoungbua P, Khow O, Rungsipipat A, Reamtong O, Sitprija V (2022). Comparative compositional and functional venomic profiles among
venom specimens from juvenile, subadult and adult Russell’s viper (Daboia
siamensis): correlation with renal pathophysiology in experimental
rabbits. J Venom Anim Toxins incl Trop Dis.

[B31] Chaiyabutr N, Vasaruchapong T, Chanhome L, Rungsipipat A, Sitprija V (2014). Acute effect of Russell's viper (Daboia siamensis) venom on renal
tubular handling of sodium in isolated rabbit kidney. Asian Biomed.

[B32] Priante G, Gianesello L, Ceol M, Del Prete D, Anglani F (2019). Cell Death in the kidney. Int J Mol Sci.

[B33] Vogler M, Braun Y, Smith VM, Westhoff M-A, Pereira RS, Pieper NM, Anders M, Callens M, Vervliet T, Abbas M, Macip S, Schmid R, Bultynck G, Dyer M JS (2025). The BCL2 family: from apoptosis mechanisms to new advances in
targeted therapy. Signal Transduct Target Ther.

[B34] Santos NA, Bezerra CS, Martins NM, Curti C, Bianchi ML, Santos AC (2008). Hydroxyl radical scavenger ameliorates cisplatin-induced
nephrotoxicity by preventing oxidative stress, redox state unbalance,
impairment of energetic metabolism and apoptosis in rat kidney
mitochondria. Cancer Chemother Pharmacol.

[B35] Liu S, Liu J, Wang Y, Deng F, Deng Z (2025). oxidative stress: signaling pathways, biological functions, and
disease. Med Comm.

[B36] Sanz AB, Sanchez-Niño MD, Ramos AM, Ortiz A (2023). Regulated cell death pathways in kidney disease. Nat Rev Nephrol.

[B37] Kamiński M, Niemczyk E, Masaoka M, Karbowski M, Hallmann A, Kedior J, Majczak A, Knap D, Nishizawa Y, Usukura J, Woźniak M, Klimek J, Wakabayashi T (2004). The switch mechanism of the cell death mode from apoptosis to
necrosis in menadione-treated human osteosarcoma cell line 143B
cells. Microsc Res Tech.

[B38] Tasoulis T, Isbister GK (2023). A current perspective on snake venom composition and constituent
protein families. Arch Toxicol.

[B39] Bregge-Silva C, Nonato MC, de Albuquerque S, Ho PL, Junqueira de Azevedo IL M, Diniz MRV, Lomonte B, Rucavado A, Díaz C, Gutierrez JM, Arantes EC (2012). Isolation and biochemical, functional and structural
characterization of a novel L-amino acid oxidase from Lachesis muta snake
venom. Toxicon.

[B40] Fox JW (2013). A brief review of the scientific history of several lesser-known
snake venom proteins: L-amino acid oxidases, hyaluronidases and
phosphodiesterases. Toxicon.

[B41] Geevarghese AV, Ranganathan H, Vishvanathan R, Benjamin PR (2025). L-amino acid oxidases from snake venom: A review of their
anticancer mechanisms and translational potential. Pharm Res Nat Products.

[B42] Du XY, Clemetson KJ (2002). Snake venom L-amino acid oxidases. Toxicon.

[B43] Oliveira IS, Pucca MB, Ferreira IG, Cerni FA, Jacob BCDS, Wiezel GA, Pinheiro- EL, Cordeiro FA, Bordon KCF, Arantes EC (2022). State-of-the-art review of snake venom phosphodiesterases
(svPDEs). Toxicon.

[B44] Chaiyabutr N, Sitprija V, Sugino N, Hoshi T (1985). Russsell’s viper venom-induced depolarization in the proximal
tubule of Triturus kidney. Thai J Vet Med.

[B45] Lossi L, Merighi A (2003). In vivo cellular and molecular mechanisms of neuronal apoptosis
in the mammalian CNS. Prog Neurobiol.

[B46] Fischer U, Schultze OK (2005). New approaches and therapeutics targeting approaches in
disease. Pharmacol Rev.

[B47] Llambi F, Green DR (2011). Apoptosis and oncogenesis: give and take in the BCL-2
family. Curr Opin Genet Dev.

[B48] Korsmeyer SJ, Shutter JR, Veis DJ, Merry DE, Oltvai ZN (1993). Bcl-2/Bax: a rheostat that regulates an anti-oxidant pathway and
cell death. Semin Cancer Biol.

[B49] Hockenbery D, Nuñez G, Milliman C, Schreiber RD, Korsmeyer SJ (1990). Bcl-2 is an inner mitochondrial membrane protein that blocks
programmed cell death. Nature.

[B50] Buttke TM, Sandstrom PA (1994). Oxidative stress as a mediator of apoptosis. Immunol Today.

[B51] Daniel NN (2007). BCL-2 family proteins: critical checkpoints of apoptotic cell
death. Clin Cancer Res.

[B52] Francesca P, Romeo G, Liu WH, Krajewski S, Reed JC, Gerhardinger C, Lorenzi M (2000). Bax is increased in the retina of diabetic subjects and is
associated with pericyte apoptosis in vivo and in vitro. Am J Pathol.

[B53] Willis SN, Fletcher JI, Kaufmann T, van Delft MF, Chen L, Czabotar PE, Ierino H, Lee EF, Fairlie WD, Bouillet P, Strasser A, Kluck RM, Adams JM, Huang DCS (2007). Apoptosis initiated when BH3 ligands engage multiple Bcl-2
homologs, not Bax or Bak. Science.

[B54] Hsu YT, Youle RJ (1998). Bax in murine thymus is a soluble monomeric protein that displays
differential detergent-induced conformations. J Biol Chem.

[B55] Lovell JF, Billen LP, Bindner S, Din AS, Fradin C, Leber B, Andrews DW (2008). Membrane binding by tBid initiates an ordered series of events
culminating in membrane permeabilization by Bax. Cell.

[B56] Green DR (2010). Means to an end: apoptosis and other cell death mechanisms.

[B57] Youle RJ, Strasser A (2008). The BCL-2 protein family: opposing activities that mediate cell
death. Nat Rev Mol Cell Biol.

